# Factors associated with prelacteal feeding in the rural population of northwest Ethiopia: a community cross-sectional study

**DOI:** 10.1186/s13006-016-0074-9

**Published:** 2016-05-25

**Authors:** Amare Tariku, Gashaw Andargie Biks, Molla Mesele Wassie, Abebaw Gebeyehu, Azeb Atinafu Getie

**Affiliations:** Department of Human Nutrition, Institute of Public Health, College of Medicine and Health Sciences, University of Gondar, Gondar, Ethiopia; Department of Health Service Management and Heath Economics, Institute of Public Health, College of Medicine and Health Sciences, University of Gondar, Gondar, Ethiopia; Department of Reproductive and Child Health, Institute of Public Health, College of Medicine and Health Sciences, University of Gondar, Gondar, Ethiopia

**Keywords:** Prelacteal feeding, Home birth, Poor knowledge of IYCF, Poor wealth status, Ethiopia

## Abstract

**Background:**

Prelacteal feeding has continued as a deep-rooted nutritional malpractice in developing countries. Prelacteal feeding is a barrier for implementation of optimal breastfeeding practices, and increases the risk of neonatal illness and mortality. However, its determinants are not well studied, which are essential to design intervention. This study aimed to assess the prevalence and determinants of prelacteal feeding among mothers with children aged 6–24 months in the rural population of northwest Ethiopia.

**Methods:**

A community based cross-sectional study was conducted in Dabat Health and Demographic Surveillance System site, Dabat district, northwest Ethiopia from May 01 to June 29, 2015. Eight hundred and twenty-two mother-child pairs were included in the study. A pretested and structured questionnaire was used to collect data. Multivariable logistic regression analysis was carried out to identify the determinants of prelacteal feeding.

**Results:**

In this community, about 26.8 % of children were given prelacteal feeds. The odds of prelacteal feeding was higher among mothers with a poor knowledge of Infant and Young Child Feeding (IYCF) (Adjusted Odds Ratio [AOR] = 3.82; 95 % Confidence Interval [CI] 2.42, 6.04), who gave birth at home (AOR = 3.74; 95 % CI 2.12, 6.60), and who were in the lowest wealth status (AOR = 2.11; 95 % CI 1.35, 3.31).

**Conclusions:**

Prelacteal feeding was common in the study area, and significantly associated with a poor household wealth status, poor maternal knowledge of IYCF, and giving birth at home. Thus, emphasis should be given to improve mothers IYCF knowledge and utilization of institutional delivery. Moreover, special attention should be given to mothers with poor socio-economic status to reduce the practice of prelacteal feeding.

## Background

Breastfeeding is unequalled way of providing ideal, natural, and renewable food for infant’s healthy growth and development [1, 2]. It provides immense immunologic, psychologic, social-economic, and environmental benefits [1, 3, 4]. Optimal breastfeeding significantly reduces a child’s risk of developing different infectious [5–8] and non-infectious inflammatory diseases [9, 10], obesity and related chronic non-communicable diseases [11–14]. Furthermore, breastfeeding promotes a child’s cognitive development [15, 16]. Breastfeeding is considered a proven child survival strategy, and the World Health Organization (WHO) recommends that, the newborns to be put on the breast within an hour after birth [1]. Early initiation of breastfeeding immediately after birth enhances mother-infant bonding [17], and creates an opportunity for the newborn to receive the nutritional and protective benefit of colostrum [3]. Moreover, it promotes effective suckling, successful establishment, and maintenance of breastfeeding throughout infancy [18].

However, in different countries including Ethiopia, a significant proportion of mothers offer prelacteal feeds to their newborn [19–25]. Prelacteal feeding increases the risk of illness from acute respiratory tract infections [8] and diarrhea [26]. Similarly, it was associated with childhood stunting [27, 28]. Furthermore, prelacteal feeding was linked with poor breastfeeding outcomes, such as higher odds of delayed initiation [24], non-exclusive [29] and early cessation [30] of breastfeeding, and delayed ‘coming in’ of breast milk [19]. Delayed initiation of breastfeeding was noted to augment the risk of neonatal mortality [31–33].

Globally, prelacteal feeding is practiced in many countries [34]; the highest rate in southeast and central Asia (54.6–93.9 %) [19, 21, 23, 24, 34] and at a modest rate in Latin America [30]. In Africa, most (10.8–75.2 %) of the mothers also offer prelacteal feeds to their newborn [20, 22, 34, 35]. In Ethiopia, about 27 % of newborns were given prelacteal feeds, and in Amhara region, the study area, half (47.8 %) of children received prelacteal feeds [27]. Though different factors were noted to affect prelacteal feeding practice, it was mainly related to maternal characteristics. Accordingly, poor household wealth status [21], older maternal age [24], maternal unemployment status, lack of education [21] and poor breastfeeding knowledge [22, 23] were associated with prelacteal feeding. The likelihood of prelacteal feeding was also higher among mothers who had no or incomplete antenatal visits [21, 36], gave birth at home [22], and delivered with cesarean section and episiotomy [23].

Since 2004, Ethiopia has implemented Infant and Young Child Feeding (IYCF) strategy as a key component of child survival strategy [37]. However, breastfeeding practice is suboptimal [27] and prelacteal feeding has been continued as a deep-rooted nutritional practice [22, 27, 38, 39]. In addition, the country has achieved a significant change in the reduction of child (under five years) mortality over the past fifteen years, but half of this mortality happened during the neonatal period [27]. Consequently, research showing the determinants of prelacteal feeding have vital importance in promoting implementation of IYCF, thereby reducing neonatal mortality. Nevertheless, literature showing the determinants of prelacteal feeding in Ethiopia, particularly northwest region are limited. Therefore, this study aimed to assess prevalence and determinants of prelacteal feeding among mothers with children aged 6–24 months in the rural population of northwest Ethiopia.

## Methods

### Study setting and design

A community-based cross-sectional study was conducted from May 01 to June 29, 2015 in Dabat Health and Demographic Surveillance System (HDSS) site which has been hosted by the University of Gondar, Gondar, Ethiopia. The site is located in the Dabat District in the northwest part of Ethiopia. The district has an estimated population size of 145,458 living in 26 rural and 4 urban kebeles *(the smallest administration unit in Ethiopia)*. The livelihood of the residents by and large depends on subsistence farming. The district has six health centers and twenty-nine health posts. The HDSS covers thirteen randomly selected kebeles (three urban and ten rural kebeles) in different ecological zones (high land, middle land, and low land) and a total of 67,385 people were living in these kebeles. The Dabat HDSS site has been running since November 1996, and collects information on vital events like birth, death, migration, and pregnancy registrations and its outcome on quarterly bases.

### Sample size and sampling procedure

Initially, the study was aimed to assess the nutritional status and feeding practice of children aged 6–59 months in Dabat HDSS site. Of the total thirteen kebeles in the HDSS, eight kebeles were selected using lottery method. Accordingly, all mothers with children aged 6–59 months who lived in the selected kebeles for at least six months were included in the study. For households with more than one study subject, only one was selected using lottery. Sample size was calculated using Epi-info version 3.7 by considering the following assumptions; the prevalence of prelacteal feeding in Amhara Region as 47.8 % [27], 95 % level of confidence, 5 % margin of error, and 5 % non-response rate. Thus, a minimum sample size of 804 was obtained. However, 822 children aged 6–24 months and fulfilling the eligibility criteria were found in the original survey. Therefore, to improve the power of the study all (822) children fulfilling the eligibility criteria were included in the study.

### Data collection tools and procedure

Data were collected using structured, pretested, and interviewer administered questionnaire. To maintain consistency, the questionnaire was first translated from English to Amharic, the native language of the study area, and was retranslated back to English by professional translators and Public Health experts. Fourteen data collectors and three field supervisors (working in Dabat DHSS) were recruited for the study. Two days intensive training regarding the objective of the study, confidentiality of information, and techniques to conduct interview was given to data collectors and supervisors. The tool was pre-tested on 5 % of the total sample out of the study area. During pre-test, the acceptability and applicability of the procedures and tools were evaluated.

### Operational definitions and study variables

Prelacteal feeding, the outcome variable, was understood as giving anything to drink other than breast milk in the first three days following delivery of the index child [40]. Accordingly, a mother was asked a key question to ascertain prelacteal feeding practice; ‘within the first three days of delivery, did you give any drink other than breast milk to the child’. If she responded “yes” it was coded ‘1’, otherwise coded ‘0’ as she didn’t give any prelacteal feed.

Mothers’ health care access was determined by asking the mother how many hours it took to reach the health care facilities. If she took less than two hours, it was considered as good health care access and was coded as “1”, otherwise “0” if she took two hours or more. Mothers’ knowledge of IYCF was assessed using nine questions. Mothers were asked about the benefit of breastfeeding, time to initiation of breastfeeding, colostrum feeding and it’s health benefit, how long an infant should exclusively breastfed, time to initiation of complementary feeding, type of food to start complementary feeding, how to feed a child, and for how long to continue on demand breastfeeding. The composite IYCF index was analyzed using Principal Component Analysis, and converted to terciles as lowest, medium, and highest. Likewise, the household wealth index was computed using a composite indicator for urban and rural residents by considering properties, like selected household assets and size of agricultural land. Principal Component Analysis was performed to categorize the household wealth index into lowest, middle, and highest.

### Data analysis

Data were entered into the EPI INFO version 3.5.3 and exported to Statistical Package for Social Sciences (SPSS) version 20 for analysis. Descriptive statistics, including frequencies and proportions were used to summarize the variables. A binary logistic regression was used to investigate factors associated with prelacteal feeding. Variables with *p*-values of < 0.2 in the bivariable analysis were entered in to the multivariable analysis. The Adjusted Odds Ratio with a 95 % confidence interval was estimated to show the strength of association, and a *p*-value of < 0.05 was used to declare the statistical significance in the multivariable analysis.

## Results

### Socio-demographic and economic characteristics

Eight hundred twenty-two mother-child pairs were included for analysis. The mean (± SD) age of children was 17.25 (±7.27) months. In the study area, substantial proportion (94 % and 89.9 %, respectively) of mothers were Orthodox Christians and married. Nearly three-quarter (69.5 %) of mothers were uneducated and 58.8 % housewives. Nearly two-thirds (63 %) of children were living with family size of more than four (Table 1).

**Table 1:** Socio-demographic and economic characteristics of study participants in the rural population of northwest Ethiopia, 2015

Variables	Frequency	Percent
Age of the child		
6–11 moths	239	29.1
12–24 months	583	70.9
Sex of the child		
Male	410	49.9
Female	412	50.1
Religion		
Orthodox	773	94.0
Other^a^	49	6.0
Mothers age		
<35	507	61.7
≥35	315	38.3
Mothers education		
Uneducated	571	69.5
Primary	109	13.3
Secondary and above	142	17.3
Mothers employment		
Housewife	482	58.6
Farmer	211	25.7
Other^b^	129	15.7
Mothers marital status		
Married	739	89.9
Currently unmarried^c^	83	10.1
Fathers education		
Uneducated	554	67.4
Primary	143	17.4
Secondary and above	125	15.2
Fathers employment		
Unemployed	46	5.6
Farmer	431	52.4
Other employment^d^	345	42.0
Household size		
≤4	304	37.0
>4	518	63.0
Wealth status		
Poor	307	37.3
Middle	246	29.9
High	269	32.7

^a^Muslim, protestant and catholic, ^b^students, unemployed, servant, own business, ^c^single, divorced and widowed, ^d^merchant, contract and permanent work

### Maternal health care related characteristics

Nearly two-third (63 %) of mothers had antenatal visits for the index child, in which about 34.4 % had greater than or equal to four visits. More than three-fourth (75.9 %) of mothers gave birth at home, and more than half (56.7 %) of their delivery was attended by relatives and volunteers. Only one-fourth (25.8 %) of mothers received postnatal care (Table 2).

**Table 2:** Health care related characteristics of mothers in the rural population of northwest Ethiopia, 2015

Variables	Frequency	Percent
Antenatal care		
No	304	37.0
Yes	518	63.0
Number of antenatal visits (*n* = 518)		
1	54	10.4
2–3	286	55.2
≥4	178	34.4
Place of delivery		
Home	624	75.9
Health facilities	198	24.1
Delivery attendant		
Health professionals	203	24.7
Traditional birth attendant	153	18.6
Relatives and volunteers	466	56.7
Postnatal care		
Yes	212	25.8
No	610	74.2
Personnel providing postnatal care (*n* = 212)		
Health Extension Worker	177	84
Other (CHW^b^ and TBA^a^)	34	16.0
Time for postnatal care started (*n* = 212)		
Within an hour	14	6.6
2–24 h	34	16
25–48 h	29	13.7
After 48 h	135	63.7
Mothers IYCF^c^ knowledge		
Poor	272	33.1
Medium	285	34.7
High	268	32.2
Health care access		
Good access	607	73.8
Poor access	215	26.2

^a^Traditional birth attendant, ^b^Community Health Worker, ^c^Infant and Young Child Feeding

### Early neonatal feeding practices

In this community, 26.8 % (95 % CI 20.9, 32.7 %) of mothers gave prelacteal feeds to their newborn. The most common prelacteal foods given were raw butter (38.6 %) and plain water (24.5 %). Nearly three-fourths (74.1 %) of mothers practiced prelacteal feeding to maintain the tradition. More than half (52.9 %) of mothers discarded colostrum; most mothers (58.4 %) perceived that it isn’t good to give to the newborn (Table 3).

**Table 3:** Early neonatal feeding practice of children (6–24 months) in the rural population of northwest Ethiopia, 2015

Variables	Frequency	Percent
Prelacteal feed given		
Yes	220	26.8
No	602	73.2
Type of prelacteal food given (*n* = 220)		
Raw butter	85	38.6
Plain water	54	24.5
Ersho^a^	21	9.6
Sugar	26	11.8
Cow milk	11	5.0
Other^b^	23	10.5
Reason for giving prelacteal food (*n* = 220)		
Tradition	163	74.1
To prevent dehydration	20	9.1
Other^c^	37	16.8
Colostrum given to the child		
Yes	435	52.9
No	387	47.1
Reason for discarding colostrum (*n* = 387)		
Not good for the child	226	58.4
Other^d^	161	41.6

^a^Ersho is a traditional baking soda prepared by heating the flour and mixing it with distilled water, ^b^fruit juice and formula milk, ^c^enriched and easier to digest than first breast milk, ^d^tradition, relatives influence, newborn feels thirsty, colostrum’s yellow and thick appearance

### Factors associated with prelacteal feeding

In the bivariable analysis, the lowest wealth status, being a housewife and a farming mother, poor maternal educational status, poor father’s educational status, poor mother’s knowledge of IYCF, and giving birth at home were significantly associated with prelacteal feeding. However the result of multivariable analysis showed that three of these, being in the lowest wealth status, having poor knowledge of IYCF and giving birth at home were significantly and independently associated with prelacteal feeding. The odds of prelacteal feeding was 3.7 times (AOR = 3.74, 95 % CI 2.12, 6.60) higher among mothers who gave birth at home. Being in the lowest wealth status increases the odds of prelacteal feeding by 2 times (AOR = 2.11, 95 % CI 1.35, 3.31). Furthermore, the higher odds of prelacteal feeding was observed among mothers with poor (AOR = 3.82, 95 % CI 2.42, 6.04) and medium (AOR = 2.22, 95 % CI 1.40, 3.54) knowledge of IYCF compared to mothers with highest knowledge of IYCF (Table 4).

**Table 4:** Factors associated with prelacteal feeding practice among mothers with children (6–24 months) in the rural population of northwest Ethiopia, 2015

Variables	Prelacteal feeding			
	Yes *n* (%)	No *n* (%)	Crude Odds Ratio (95 % CI)	Adjusted Odds Ratio (95 % CI)
Mother’s marital status				
Currently married	203 (27.5)	536 (72.5)	1	
Currently unmarried	17 (20.5)	66 (79.5)	0.68 (0.39, 1.19)	1.09 (0.51, 2.33)
Mother’s education				
Uneducated	162 (28.4)	409 (71.6)	2.91 (1.70, 4.99)^*^	1.19 (0.59, 2.39)
Primary	41 (37.6)	68 (62.4)	4.43 (2.34, 8.39)^*^	1.97 (0.92,4.25)
Secondary and above	17 (12)	125 (88)	1	1
Mother’s employment				
Housewife	134 (27.8)	348 (72.2)	2.72 (1.56, 4.76)^*^	1.85 (0.94, 3.64)
Farmer	70 (33.2)	141 (66.8)	3.51 (1.93, 6.37)^*^	1.39 (0.68, 2.84)
Other	16 (12.4)	113 (87.6)	1	1
Father’s education				
Uneducated	144 (26)	410 (74)	3.03 (1.65, 5.54)^*^	1.15 (0.53, 2.51)
Primary	63 (44.1)	80 (55.9)	6.785 (3.49, 13.16)^*^	3.26 (0.98, 7.32)
Secondary and above	13 (10.4)	112 (89.6)	1	1
Mother’s knowledge of IYCF^a^				
Poor	102 (37.5)	170 (62.5)	3.28 (2.17, 4.96)^*^	3.82 (2.42, 6.04)^*^
Medium	77 (27)	208 (73)	2.02 (1.32, 3.09)^*^	2.22 (1.39, 3.54)^*^
High	41 (15.5)	224 (84.5)	1	1
Wealth status				
Poor	118 (38.4)	189 (61.6)	2.80 (1.91, 412)^*^	2.11 (1.35, 3.31)^*^
Medium	53 (21.5)	193 (79.5)	1.23 (0.79, 190)	0.83 (0.50, 1.38)
High	49 (18.2)	220 (81.8)	1	1
Mother’s age				
<35	124 (24.5)	383 (75.5)	1	1
≥35	96 (30.5)	219 (69.5)	1.35 (0.99, 1.85)	1.41 (0.98, 2.04)
Place of delivery				
Home	202 (32.4)	422 (67.6)	4.79 (2.87, 7.99)^*^	3.74 (2.12, 6.60)^*^
Health facility	18 (9.1)	180 (90.9)	1	1

^*^ *p* < 0.05, ^a^Infant and Young Child Feeding

## Discussion

This study revealed that, the prevalence of prelacteal feeding was 26.8 %. The finding was in agreement with the 2011 Ethiopian Demographic and Health Survey report of 27 % [27]. Slightly higher prelacteal feeding was reported in Raya Kobo district, Ethiopia at 38.8 % [22]. This is probably related to similarities in the lower maternal health care utilization, lower rate of an institutional delivery, and low maternal educational status in the study areas. The magnitude of prelacteal feeding was also high in some countries where most of the mothers were more educated, such as Egypt (57.8 %), Nigeria (70.6 %) [41] and Vietnam (73.3 %) [23]. This might be due to mothers perceived delay in breast milk production, insufficient milk production, and the perceived risk of dehydration and hypoglycemia to the newborns. Other studies also claimed that prelacteal feeding practice was associated with mothers’ unfavorable attitude towards it [42, 43]. However, behavioral change interventions have had a profound effect in reducing prelacteal feeding and other inappropriate breastfeeding practices and IYCF recommendations [20]. Thus, such evidence could further necessitate incorporating behavioral change and communication components in IYCF counseling and formal education curricula (health and medical education curricula).

The odds of prelacteal feeding were higher among mothers with poor and medium knowledge of IYCF as compared to their counterparts with a higher IYCF knowledge. The finding was supported by other study reports [22, 23]. Boosting a mothers knowledge of IYCF is cornerstone for implementing sustainable strategies to improve appropriate feeding practices [44].

In line with other study findings in Ethiopia [22, 27], this study also showed that, home birthing also increases prelacteal feeding. This is could be due to the fact that mothers who gave birth at home, were more likely to be exposed to the traditional beliefs that favor prelacteal feeding. In contrast, attending an institutional delivery would have an added benefit to receive immediate obstetric care, such as early initiation of breastfeeding which reduces the likelihood of giving prelacteal feeding [22]. However, other studies claimed that, giving birth through cesarean-section was associated with prelacteal feeding, where mainly formula milk was given until initiation of breastfeeding [23, 24, 45].

In this study, poor wealth status was strongly associated with prelacteal feeding. The parallel finding was reported by the Ethiopian Demographic and Health Survey report [27], in which the odds of prelacteal feeding declines with increasing the household wealth status. This could be related to the positive effect of wealth in improving mother’s health seeking behavior and the utilization of maternal health care services, which is the main service delivery point to promote clients awareness on IYCF. In Ethiopia, mothers in the lowest socio-economic status were less likely to have antenatal care and attend institutional delivery [27]. However, the finding is not in agreement with the study results elsewhere [21, 45]. The discrepancy may be attributed to the expensiveness of prelacteal food in the later study areas as compared to the current study area. Accordingly, being in the highest wealth status will enable people to purchase expensive formulae to feed their newborn prior to the initiation of breastfeeding compared with people in the lowest wealth status.

The study tried to show pertinent determinants of prelacteal feeding practice in the rural population of northwest Ethiopia by using an optimal sample size. However, the study is not free from some of the limitations. Though we have tried our best to maintain the quality of the data, there might be a chance of recall bias and information bias in ascertaining some of the variables, such as health care access and time to initiation of postnatal care.

## Conclusions

In summary, prelacteal feeding was common in the study area, and significantly associated with poor household wealth status, poor maternal knowledge of IYCF, and giving birth at home. Thus, emphasis should be given to improve mothers’ IYCF knowledge and utilization of institutional delivery. Moreover, special attention should be given to mothers with poor socio-economic status to reduce prelacteal feeding practice.

## Abbreviations

AOR: adjusted odds ratio
CI: confidence interval
COR: crude odds ratio
HDSS: Dabat Health and Demographic Surveillance System
IYCF: infant and young child feeding
SD: standard deviation
SPSS: statistical package for social sciences
WHO: World Health Organization
